# The Effect of the California Tobacco Control Program on Smoking Prevalence, Cigarette Consumption, and Healthcare Costs: 1989–2008

**DOI:** 10.1371/journal.pone.0047145

**Published:** 2013-02-13

**Authors:** James Lightwood, Stanton A. Glantz

**Affiliations:** 1 School of Pharmacy and Center for Tobacco Control Research and Education, University of California San Francisco, San Francisco, California, United States of America; 2 Department of Medicine (Cardiology), Center for Tobacco Control Research and Education, and Philip R. Lee Institute for Health Policy Studies, University of California San Francisco, San Francisco, California, United States of America; The University of Hong Kong, Hong Kong

## Abstract

**Background:**

Previous research has shown that tobacco control funding in California has reduced per capita cigarette consumption and per capita healthcare expenditures. This paper refines our earlier model by estimating the effect of California tobacco control funding on current smoking prevalence and cigarette consumption per smoker and the effect of prevalence and consumption on per capita healthcare expenditures. The results are used to calculate new estimates of the effect of the California Tobacco Program.

**Methodology/Principal Findings:**

Using state-specific aggregate data, current smoking prevalence and cigarette consumption per smoker are modeled as functions of cumulative California and control states' per capita tobacco control funding, cigarette price, and per capita income. Per capita healthcare expenditures are modeled as a function of prevalence of current smoking, cigarette consumption per smoker, and per capita income. One additional dollar of cumulative per capita tobacco control funding is associated with reduction in current smoking prevalence of 0.0497 (SE.00347) percentage points and current smoker cigarette consumption of 1.39 (SE.132) packs per smoker per year. Reductions of one percentage point in current smoking prevalence and one pack smoked per smoker are associated with $35.4 (SE $9.85) and $3.14 (SE.786) reductions in per capita healthcare expenditure, respectively (2010 dollars), using the National Income and Product Accounts (NIPA) measure of healthcare spending.

**Conclusions/Significance:**

Between FY 1989 and 2008 the California Tobacco Program cost $2.4 billion and led to cumulative NIPA healthcare expenditure savings of $134 (SE $30.5) billion.

## Introduction

Previous research using aggregate state level data found a relationship between per capita funding for population-based tobacco control programs and reductions in per capita cigarette consumption, which were in turn associated with reductions in per capita healthcare costs in California [1]. These estimates are consistent with those found in a subsequent independent study [2] that estimated the average effect of tobacco control expenditures across states.

The California Tobacco Control Program was established in 1989. It adopted a comprehensive approach designed to change social norms to reinforce the nonsmoking norm rather than a frontal attack on smokers that markets cessation services. The social norm change approach seeks to indirectly influence current and potential future tobacco users by creating a social milieu and legal climate in which tobacco becomes less desirable, acceptable and accessible. The Program combines an aggressive media campaign with three consistent themes (the tobacco industry lies, nicotine is addictive, and secondhand smoke kills) with public policy change, particularly in the area of promoting smokefree environments. The Program has been premised on the fact that youth smoking will decline when more adults stop smoking [1].

Per capita cigarette consumption, which includes all the nonsmokers, is a very simple measure of smoking behavior. Tobacco control program funding may affect smoking prevalence and cigarette consumption per current smoker differently, and each, in turn, may have a different effect on healthcare expenditures. This paper refines our earlier model by replacing total per capita consumption with a two-dimensional measure of smoking behavior – prevalence of current smoking and cigarette consumption per smoker. This two dimensional measure may provide more insight into the mechanisms by which tobacco control programs work and how reductions in smoking reduces healthcare expenditures and may provide a better predictive model for use in forecasting the effect of policy changes on smoking and healthcare expenditure.

The estimates for the new model, which are based on a different sample period than the old model (due to limits on state specific data on prevalence), show that increased per capita cumulative tobacco control funding is associated with reductions in both prevalence and cigarette consumption per smoker, and reductions in both measures of smoking behavior reduce per capita healthcare expenditures in California compared to control states. Newly available data for a commonly used measure of healthcare expenditure from the Centers for Medicare and Medicaid Services allowed a true out of sample forecasting experiment; the new model using prevalence and average cigarette consumption per smoker produces better forecasts than the previously published per capita cigarette consumption model [1].

## Methods

As in our earlier work [1], this analysis compares smoking behavior and healthcare time series variables for California with those for an aggregate population from thirty-eight control states that did not have substantial state tobacco control programs or cigarette tax increases of more than $0.50 before the year 2000 [3]. See our earlier paper [1] for details on the selection of control states and justification for using cumulative per capita control spending as the independent variable.

### Model

The new model consists of three equations: one equation for the relationship between cumulative per capita tobacco control funding and current smoking prevalence; one for the relationship between tobacco control funding and cigarette consumption per smoker; and one for the relationship between smoking behavior (prevalence of smoking and cigarette consumption per smoker) and per capita healthcare expenditures.

#### Current Smoking Prevalence



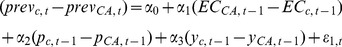
(1)
*Cigarette Consumption per Smoker:*

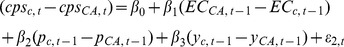
(2)


*Current Smoking Prevalence, Cigarette Consumption per Smoker and Healthcare Expenditures:*

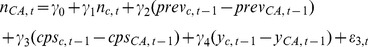
(3)Where 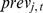: Prevalence of current smoking in population *j*, for California and control states in year *t*, in percentage points, 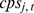: Cigarette consumption per current smoker in population *j*, for California and control states in year *t*, in packs/year per smoker, 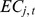: Cumulative per capita tobacco control funding in population *j*, for California and control states in year *t*, in dollars, 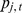: Price per pack of cigarettes in population *j*, for California and control states in year *t*, in dollars, 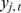: Per capita personal income in population *j*, for California and control states in year *t*, in thousands of dollars, 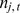: Per capita healthcare expenditures in population *j*, for California and control states in year *t*, in thousands of dollars, 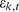: Stationary error terms for equation *k* = 1 to 3, in year *t*, 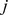: Index for population 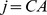 for California (intervention), *j* = *c* for control state populations, *t*: Time index, t = 1985 to 2008 (24 annual observations).

All monetary values are expressed in year 2010 dollars using the Medical Care (healthcare expenditures) and All-Item (tobacco control funding, cigarette price and personal income) Consumer Price Index for Urban Consumers (CPI-U) [4]. Nominal dollars were converted to 2010 dollars using the Bureau of Labor Statistics CPI-U indices for each Census Region using the relevant Census Region price index [4]. State cigarette sales were used to aggregate individual control state average cigarette sales prices; population weights were used to aggregate the remaining control state variables.

Equation 1 explains the difference between current smoking prevalence in the control states and California 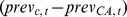 as a function of the corresponding differences between cumulative per capita tobacco control funding 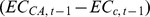, cigarette price 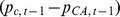 and per capita personal income 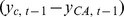. Equation 2 explains the difference between control states and California cigarettes consumed per current smoker 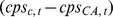 as a function of the same explanatory variables as Equation 1. Equation 3 explains per capita health expenditures in California 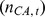 as a function of per capita healthcare expenditures in the control states 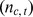, and the differences between California and control states' current smoking prevalence 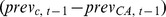, cigarette consumption per smoker 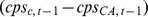 and real personal per capita income 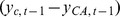.

Equations 1 to 3 are generalizations of the model estimated in previous research for California [1]. The major change from the previous model is that prevalence of current smoking and cigarette consumption per smoker constitute a two-dimensional measure of smoking behavior rather than the single dimension of per capita cigarette consumption. There are two additional modifications, based on related research on Arizona [5]: we use the difference in price between the control states and California (i.e., require that the sum of the price coefficients for the control states and California sum to zero) and we added the variables for income. (See description of statistical analysis below for details).

From published research on per capita cigarette consumption, we expect that cigarette consumption per current smoker (Equation 2) will be negatively related to per capita tobacco control funding [6], [7] and the price of cigarettes [8], [9]. Previous time series estimates have shown cigarette consumption to be positively related to measures of per capita income [8]. We found one publication with aggregate time series regression estimates for prevalence of smoking (Equation 2), which found a negative price elasticity and a positive elasticity for per capita income, and mixed results for tobacco control funding [10]. Cross-sectional estimates based on individual survey responses show a positive relationship between prevalence and income for lower income individuals, which is consistent with aggregate time series estimates if the effect of income changes among lower income individuals dominates that of higher incomes over time [11]. Per capita healthcare expenditure for California should be positively related to per capita healthcare expenditure for the control states (reflecting common trends in advances in medical technology) and income [12]. Over time, per capita healthcare expenditure may or may not be positively related to smoking behavior; the sign will be determined by whether the effect of lower expenditures associated with less smoking in a population of fixed size is greater than higher expenditures due to longer lived non-smokers and smokers who consume fewer cigarettes [13].

### Data

Consumption per smoker was calculated by dividing per capita cigarette consumption for the respective populations by current smoking prevalence. The definition of tobacco control funding used for the main analysis included state and federal funding; private funding was omitted, though including it makes almost no difference in the results. Cumulative real per capita tobacco control funding was constructed by summing annual real per capita funding.

The main results use the National Income and Product Account (NIPA) measure of per capita healthcare expenditure. Sensitivity analyses used an alternative measure of healthcare expenditure from the Centers for Medicare and Medicaid Services (CMS) [14], [15] that was used in our earlier work [1]. The NIPA and CMS measures differ mainly in that the former omits items such as medical equipment, prescription drugs, administrative expenditures and insurance premiums [16]. The two measures are highly correlated over time, and both include expenditures for hospital services, medical procedures and healthcare personnel [16].

Per capita healthcare expenditures were calculated by dividing totals by the state resident populations. For sensitivity analysis the population was adjusted for race (African-American, white and other) and ethnicity (Hispanic and non-Hispanic).

The sample for the model connecting per capita tobacco control funding to smoking behavior consists of 24 annual observations from 1985 to 2008 (The 1984 observation was lost due to lagging the explanatory variables one period).

The 38 control states are Alabama, Arkansas, Colorado, Connecticut, Delaware, Georgia, Idaho, Illinois, Indiana, Iowa, Kansas, Kentucky, Louisiana, Maine, Minnesota, Mississippi, Missouri, Montana, Nebraska, Nevada, New Hampshire, New Mexico, North Carolina, North Dakota, Ohio, Oklahoma, Pennsylvania, Rhode Island, South Carolina, South Dakota, Tennessee, Texas, Utah, Vermont, Virginia, West Virginia, Wisconsin, and Wyoming.

Estimates of smoking prevalence are not available for all of the 38 control states starting in 1985; data from 13 states were available as of 1984 and all were available by 1994. As a result, each of the 38 control states contributed to the control population as annual estimates of state smoking prevalence became available.

See the online Supporting Information S1 for all data sources and additional details of variable construction.

### Statistical Analysis

The variables were tested to determine whether they were stationary or nonstationary. The main statistical analysis used a regression specification called a reduced form vector autoregression (VAR) in which the explanatory variables are expressed as a function of the lagged explanatory variables. The reduced form VAR can be used for unbiased estimates regardless of whether the data are stationary or nonstationary [17], [18]. As reported in the Results section, it was difficult to determine whether smoking prevalence was stationary or nonstationary, therefore the reduced form VAR was the most robust approach to estimation.

Equations 1 to 3 were estimated using an instrumental variables technique that assured that bias would not result from correlation between the explanatory variables and the regression error terms in Equations 1 to 3; the instrumental variables did not use observed data, but were calculated using a formula that produces the required properties for unbiased estimation when the data are nonstationary [19], [20]. The regression coefficient standard errors were estimated using a robust technique to guard against bias due to violations of the usual assumptions on regression errors [19], [20], [21]. The regression residuals were tested to determine whether they were stationary or nonstationary; if the regression errors are nonstationary then the regression coefficients may not be consistent, and may indicate associations when the variables are actually independent [18]. The behavior of the regression residuals was checked for normality, serial correlation, heteroskedasticity, influential outliers and structural breaks [22].

See the online Supporting Information S1 for additional details on the statistical analysis.

Oracle Crystal Ball [23], OxMetrics 6.10 [24] and Stata version 12.0 [25] were used for estimation.

### Estimated Program Effect

The effect of the California Tobacco Control Program was estimated using model predictions of the historical time series and predictions of a counterfactual history with all California tobacco control funding set to zero from FY1989 through FY2008. Monte Carlo simulations, using the regression results, estimated the effect of the California Tobacco Control Program. Predictions for prevalence (Equation 1) and consumption per smoker (Equation 2) were used as explanatory variables in the per capita healthcare expenditure model (Equation 3) instead of observed values. The dependent variables in Equations 1 and 2 are expressed as differences between California and control states; predictions of California prevalence and cigarette consumption per smoker were calculated by subtracting the corresponding observed control state values from the predicted difference between California and the control states. The total reduction in prevalence of smoking, person years of smoking, cigarette consumption per smoker, value of lost sales of cigarettes to the tobacco companies, and reduction in healthcare expenditure and other statistics were calculated by subtracting the difference between the model predictions using historical California tobacco control funding and predictions with the history of funding set to zero.

### Sensitivity Analysis

Several sensitivity analyses were conducted to check the robustness of the methods and estimation results. See the Online Supporting Information S1 for additional details.

#### Validation of model specification using a specification search algorithm

It may be difficult to determine the best specification of a regression with a relatively small sample (up to 24 annual observations in this study). Therefore an automatic model selection algorithm, the Autometrics module in Oxmetrics [22], was used to explore the robustness of the regression specification and validate the adequacy of Equations 1 to 3. Autometrics [22] chooses the best model specification from a list of explanatory variables in a way that preserves the validity of the final estimates of standard errors of the regression coefficients, and therefore validity of the significance level for hypothesis tests on the coefficients. Autometrics also screens regression specifications for acceptable performance of regression residuals.

#### Use of alternative estimators

If the data are nonstationary, then the estimates using the VAR specification should be consistent with those from a static regression (called a “cointegrating regression”) [17], [26], using either an ordinary least squares or instrumental variables estimates. The coefficients in the static specification represent the long run relationship between the explanatory and dependent variables, while the coefficients from the VAR specification contain information about the long run relationship and the short run adjustment process [18]. In this sensitivity analysis Equations 1 to 3 were re-estimated using a static regression using the same instrumental variables estimator used for the main analysis, ordinary least squares, and robust regression in order to compare for consistency with the reduced form VAR results.

The prevalence (Equation 1) and cigarette consumption per smoker (Equation 2) regressions were also re-estimated assuming that the variables were stationary and that there was exponential decay in the effect of annual tobacco control funding on smoking behavior in order to explore alternatives to the assumption that there was no detectable decay in effectiveness of annual tobacco control expenditures over the sample period.

#### Alternative Selection of control states

The model was estimated using different groups of control states to explore the sensitivity of the results to control states that would reflect different regional trends in the explanatory variables, particularly healthcare expenditure and smoking behavior.

#### Alternative specification for consumption per smoker

The automatic selection procedure, Autometrics, used to check the specifications of Equations 1 to 3, found an alternative specification for Equation 2 (cigarette consumption per smoker) that was also acceptable and nearly equivalent by the selection criterion. The analysis was redone using this alternative regression model for cigarette consumption per smoker (Equation 2).

#### Race and Ethnicity

The model was re-estimated with variables for racial and ethnic composition of California and control populations, using estimates of the proportion of Hispanic, Black and All Other races from BRFSS survey data, added to the Equations 1 to 3 in order to determine the sensitivity of the regression estimates to these population characteristics.

#### Including private tobacco control funding

The model was estimated with alternate measures of tobacco control funding that included private nonprofit funding.

### Estimates using Centers for Medicare and Medicaid Services (CMS) Healthcare Expenditure Data

The CMS provides a commonly used measure of healthcare expenditure for the U.S. and individual states, though state specific estimates are not released at regular intervals. CMS healthcare expenditure data were used to estimate Equation 3 for the sample periods 1984 to 2004 that was used in our previous research [1] in order to check robustness of the results to different measures of healthcare expenditure and to estimate results for total healthcare expenditures. The CMS measure of healthcare expenditure is denoted by 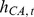 (for California) and 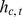 (for control states) to distinguish it from the NIPA measure (which is denoted by 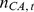 for California and 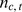 for control states). Program effects were calculated using the estimates to determine whether the results of the new model were consistent with those of the old model. Estimates for 1984 to 2008 and program effects were calculated.

### Out of sample forecasts of the CMS measure for healthcare

CMS healthcare expenditure data (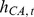 and 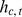) for the years 2005 to 2008 became available during December, 2011, after the other analysis presented in this paper was completed. We used these additional data to compare the out-of-sample forecast performance of the old model (that used per capita cigarette consumption) and the new model (that used smoking prevalence and cigarette consumption per smoker). We re-estimated the model from our previous research that used per capita cigarette consumption as the measure of smoking behavior (Equations 1 and 2 in [1]), and Equations 1 to 3 in the new model presented in this study using prevalence and cigarette consumption per smoker, using a similar sample period (years 1984 to 2004) to that in the earlier paper, and using the reduced form VAR specification. We calculated forecasts for per capita cigarette consumption, per capita healthcare expenditure, and four measures of forecast accuracy (root mean square error, mean absolute error, mean absolute percentage error, and the regression slope coefficient of the forecast on observed values) for the years 2005 to 2008 to compare the forecast performance of the two models (Table S1, Supporting Information S1).

## Results

### Time Series Properties of the Variables

The unit root tests indicated that all the variables except for prevalence of current smoking were nonstationary with autoregressive unit roots; the results for prevalence were unstable and difficult to interpret. Smoking prevalence may be stationary, so estimation using cointegrating regressions (which were used in previous research) may be inappropriate. These results imply that that the reduced form VAR specification is more robust than the cointegrating regression estimates (used in earlier research [1], [5]) since the VAR can be used with both stationary or nonstationary data.

### Model Estimates

The reduced form VAR estimates of Equations 1 and 2 show statistically significant associations between cumulative per capita tobacco control funding and both measures of smoking behavior (prevalence and cigarette consumption per smoker). Holding other variables constant, an additional dollar in cumulative per capita California tobacco control funding reduces California prevalence by 0.0497 (SE 0.00347; P<0.01) percentage points and reduces cigarette consumption per smoker by 1.39 (SE 0.132; P<0.01) packs/year. Equation 3 shows statistically significant associations between and between both measures of smoking behavior and per capita healthcare expenditures (Table 1). A one percentage point reduction in smoking prevalence and one pack/year reduction in cigarette consumption per smoker in California reduces per capita healthcare expenditures by $35.4 (SE $9.85) (P<0.01) and $3.14 (SE $0.786; P<0.01), respectively (Table 1).

**Table 1:** Estimated California smoking prevalence, cigarettes per capita, and per capita healthcare expenditures.

Eq.	Sample Period	Dependent Variable	Statistic	Estimate	dimension
1	1985–2008, 24 obs	(*prev_c, t_ – prev_CA, t_*)	*α_0_*	6.30 (0.610)	
			*α_1_*	0.0497 (0.00347)	/$ per capita
			*α_2_*	−1.00 (0.477)	/$ per pack
			*α_3_*	0.416 (0.0730)	/$1000 per capita
			*R^2^* (%)	77	
			*r_1_*	0.154	
2	1985–2008, 24 obs	(*cps_c, t_ – cps_CA, t_*)	*β_0_*	67.9 (10.2)	
			*β_1_*	1.39 (0.132)	/$ per capita
			*β_2_*	−26.6 (6.80)	/$ per pack
			*β_3_*	2.97 (1.21)	/$1000 per capita
			*R^2^* (%)	81	
			*r_1_*	0.148	
3	1985–2008, 24 obs	*n_CA, t_*	*γ_0_*	−550 (433)	$
			*γ_1_*	1.15 (0.180)	
			*γ_2_*	−35.4 (9.85)	$/%point
			*γ_3_*	−3.14 (0.786)	$ pack per smoker
			*γ_4_*	−108 (6.79)	$/$1000 per capita
			*R^2^* (%)	80	
			*r_1_*	0.262	
3*	1985–2008, 24 obs	*h_CA, t_*	*γ_0_*	1056 (112)	$
			*γ_1_*	0.847 (0.0542)	
			*γ_2_*	−67.8 (7.31)	$/%point
			*γ_3_*	−5.48 (0.928)	$ pack per smoker
			*γ_4_*	−107 (22.3)	$/$1000 per capita
			*R^2^* (%)	89	
			*r_1_*	0.486†	
3*	1985–2004, 20 obs	*h_CA, t_*	*γ_0_*	1001 (967)	$
			*γ_1_*	0.856 (0.227)	
			*γ_2_*	−69.8 (12.6)	$/%point
			*γ_3_*	−5.59 (1.77)	$ pack per smoker
			*γ_4_*	−112 (17.5)	$/$1000 per capita
			*R^2^* (%)	78	
			*r_1_*	0.483†	

* Equation 3 with *h_CA, t_* as dependent variable instead of *n_CA, t_* and *h_c, t_* as explanatory variable instead of *n_c, t_*.

† significant at the 5% level.

*r_1_*: first order autocorrelation coefficient.

*prev_j, t_*: Prevalence of current smoking in population *j*, for California and control states in year *t*,(percentage points).

*cps_j, t_*: Cigarettes consumption per current smoker in population *j*, for California and control states in year *t*, (packs/year per smoker).

*EC_j, t_*: Cumulative per capita funding in population *j*, for California and control states in year *t*, (dollars).

*p_j, t_*: Price per pack of cigarettes in population *j*, for California and control states in year *t*, (dollars).

*y_j, t_*: Per capita personal income in population *j*, for California and control states in year *t*, (thousands of dollars).

*n_j, t_*: Per capita healthcare expenditures in population *j*, for California and control states in year *t*, (thousands of dollars).

*h_j, t_*: Per capita healthcare expenditures in population *j*, for California and control states in year *t*, (thousands of dollars).

All of the other explanatory variables are statistically significant at the one percent level except the price of cigarettes (*α*_2_) in Equation 1 (P = 0.049) and per capita income (*β*_3_) (P = 0.023) in Equation 2 (Table 1). The signs of the other explanatory variables are as expected according to economic theory and previous research: prevalence and cigarette consumption per smoker were negatively related to cigarette price. Cigarette consumption per smoker is positively related to per capita income which is consistent with existing time series and addictive models for consumption [2], [8], [9], [27]. Per capita healthcare expenditure is positively associated with per capita income. The residuals show no violations of assumptions that would affect the interpretation of the regression estimates.

**Figure 1:**
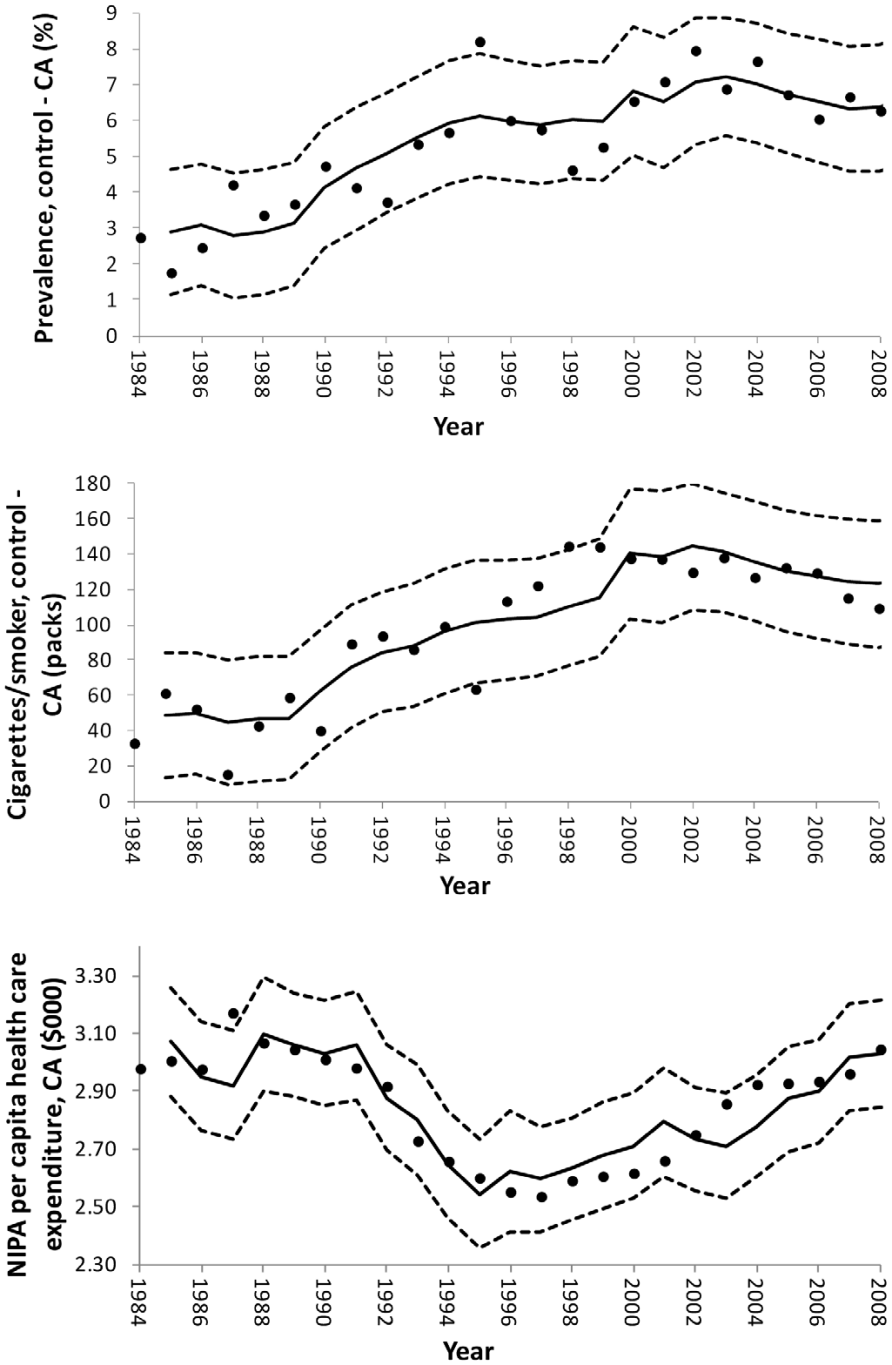
Observed and predicted smoking prevalence, cigarette consumption per smoker and per capita healthcare expenditures. Top panel: Difference between California and control state current smoking prevalence (Equation 1), middle panel: difference between California and control state cigarette consumption per smoker (Equation 2), bottom panel: California per capita healthcare expenditures using the NIPA measure (Equation 3). Black circles: observed, solid line: in-sample predictions from regression estimates, dashed lines: 95 percent forecast confidence intervals for prediction of individual observations.

The in-sample predictions for prevalence (Equation 1) and healthcare expenditure (Equation 3) show good agreement with the observed data (Figure 1). Cigarette consumption per smoker (Equation 2) does not seem to model turning points in the data well, though it is a better model for longer run trends (Figure 1).

### Tobacco Control Program Effect

**Figure 2:**
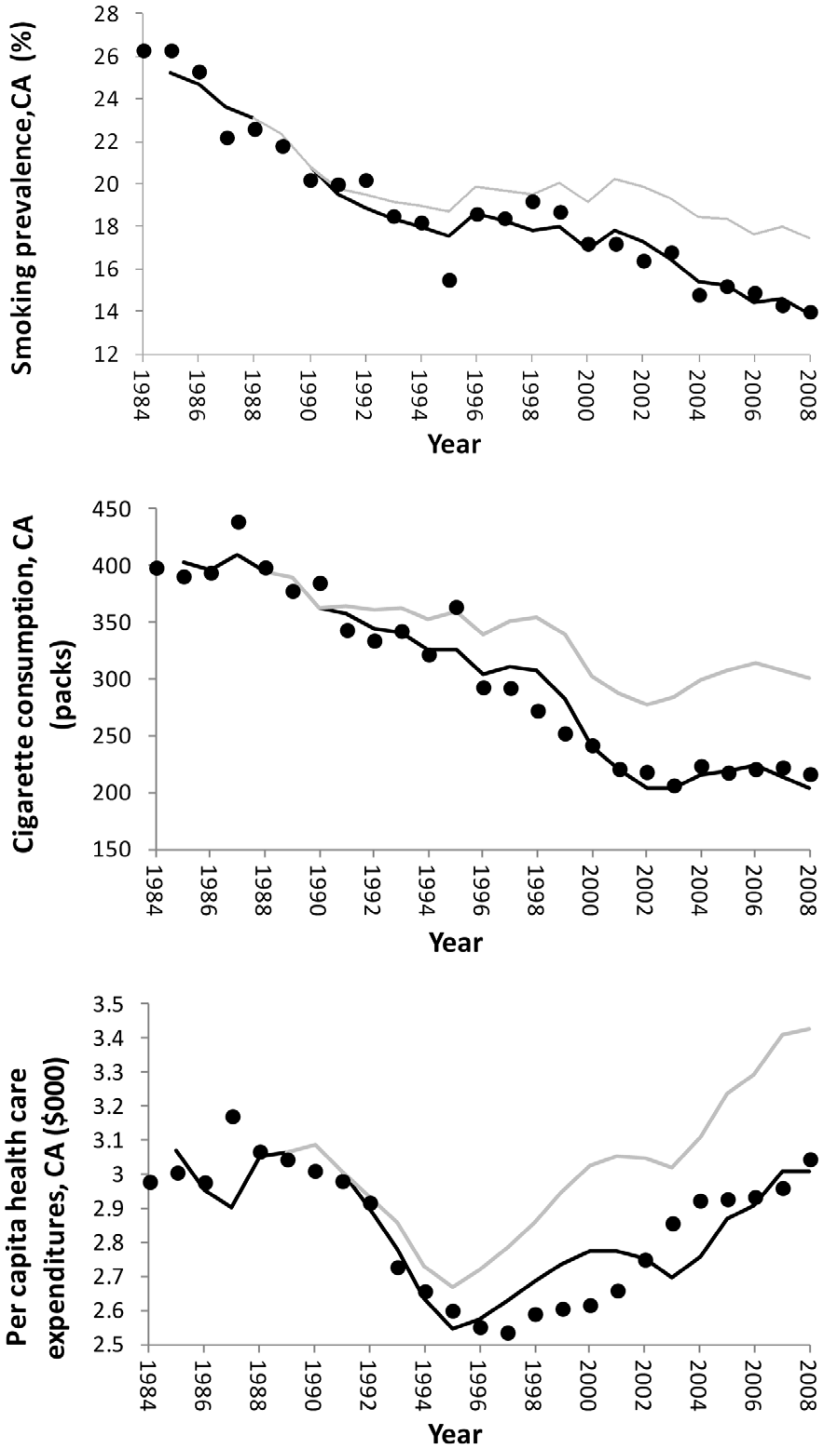
Prevalence of current smoking, cigarette consumption per smoker and per capita healthcare expenditures with and without California tobacco control funding, Top panel: California current smoking prevalence, middle panel: California cigarette consumption per smoker, bottom panel: California per capita healthcare expenditures using the NIPA measure. Black circles: observed, black line: predictions with California tobacco control program (using historical data on tobacco control funding), gray line: predictions without California tobacco control program (California tobacco control funding set to zero).

The dynamic simulation of the time paths of prevalence of smoking, consumption per smoker and per capita healthcare expenditures (Figure 2) is similar to those for the in-sample fits for Equations 1 to 3. The reductions in prevalence, cigarette consumption per smoker and per capita healthcare expenditure attributable to the Program increase steadily beginning in FY 1992 (Figure 2).

In fiscal year 2008, 19 years after the Program started, smoking prevalence was 3.46 (SE 0.242) percentage points and cigarette consumption per smoker was 96.3 (SE 13.7) packs/year, and per capita healthcare expenditures were $411 (SE $92.0) below what is predicted in the absence of the California Tobacco Control Program.

From FY1989 to FY2008, the Program is associated with a cumulative reduction in 8.79 (SE 0.616) million person-years of smoking, 6.79 (SE 0.605) billion packs of cigarettes worth $28.5 (SE $2.55) billion in pre-tax sales to the cigarette companies. The cumulative savings in the NIPA measure of healthcare expenditures is $134 (SE $30.5) billion for the years 1989 to 2008.

The reduction in prevalence is responsible for 36.4% (SE 4.06%) of the reduction in cumulative total cigarette consumption per smoker and 31.2% (SE 3.48%) of the reduction in NIPA healthcare expenditures, respectively. The rest of the reductions are due to reductions in consumption per smoker.

See online Supporting Information S1 for the additional details of calculation of the tobacco control program effect.

### Sensitivity Analysis

#### Validation of model specification using a specification search algorithm

Autometrics selected regression specifications are similar to those for prevalence (Equation 1) and per capita healthcare expenditure (Equation 3) and the algorithm found no competing specifications that substantially changed the coefficient values for per capita tobacco control funding (Equation 1) or prevalence and cigarette consumption per smoker (Equation 3).

Autometrics did select a regression specification for Equation 2 that contained only California and control tobacco control funding variables and California cigarette price when the variables were entered individually. This alternative specification produces a statistically significant relationship between California tobacco control funding and cigarette consumption per smoker. However, this alternative specification results in very large estimates of program effects because it does not include the effect of common trends represented by variables for control states (such as cigarette consumption per smoker), therefore the initial specification was chosen to produce lower estimates of program effect.

#### Alternative estimators and control states

The results of the OLS and robust regression estimates of the VAR and cointegrating regressions are consistent with those of the reduced form VAR estimates and the residuals are stationary. This result provides more evidence that data are nonstationary and that the results are robust to different regression specifications.

Models that estimated an exponential decay in the effect of tobacco control did not produce statistically significant regression relationships and the residuals showed significant autocorrelation.

#### Alternative Selection of Control States

The estimates for Equations 1 to 3 using alternative control populations are similar to the main results. Estimates for all the alternative groups of control states show statistically significant relationships between California tobacco control funding and both prevalence and cigarette consumption per smoker and between those measures of smoking behavior and per capita healthcare expenditure. The principal difference is for the healthcare expenditure (Equation 3): when the Western states were used as controls, the coefficient for consumption per smoker is $0.92 (SE $0.283) which is significantly different and lower than in the main analysis (P = 0.011).

#### Alternative specification of consumption per smoker

The estimated coefficients of the alternative model chosen by Autometrics are −2.96 (SE 0.232) for the difference California and control state tobacco control funding and −15.46 (SE 5.00) for the price of cigarettes in California. Tobacco control funding has a statistically significant effect on cigarette consumption per smoker in California in the alternative model.

#### Race and Ethnicity

The variables for proportion of the population that African-American or Hispanic do not enter the regressions (all P values>0.10) and their inclusion do not change the values of the other coefficients substantially. The variable for Other Race (neither White nor African-American) enter the regressions for prevalence (Equation 1) and cigarettes consumption per smoker (Equation 2) at the 5 percent significance level with a positive sign for prevalence and a negative sign for consumption per smoker. California Tobacco control funding is more effective holding the prevalence of Other Races constant, implying that tobacco control funding is less effective in Other Races than the rest of the population.

### Centers for Medicare and Medicaid Services (CMS) Healthcare Expenditure

**Figure 3:**
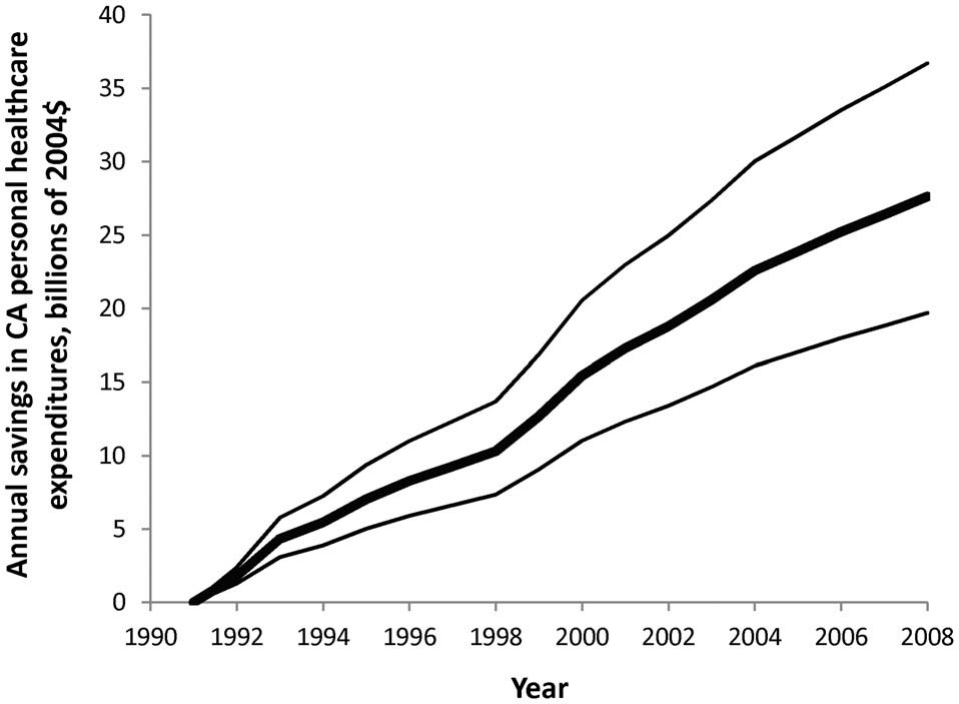
Annual savings in total personal healthcare expenditures in California attributable to the California Tobacco Control Program, billions of 2010 dollars.

Estimates of healthcare expenditure using the CMS measure of healthcare expenditure (rather than the NIPA measure) from 1989 to 2004 show a reduction of one percentage point in prevalence of current smoking and consumption of one pack per year per smoker in California reducing per capita healthcare expenditures by $69.8 (SE $12.6) and $5.59 (SE $1.77), respectively (Table 1). The California Tobacco Program is associated with a cumulative reduction of $142 (SE $22.4) billion in CMS healthcare expenditures between 1989 and 2004. Estimates of healthcare expenditure using the CMS measure of healthcare expenditure (rather than the NIPA measure) from 1989 to 2008 show that reductions of one percentage point in prevalence of current smoking and in consumption of one pack per year per smoker in California reduce per capita healthcare expenditures by $67.8 (SE $7.31) and $5.48 (SE $0.928), respectively (Table 1). The California Tobacco Control Program is associated with a steady increase in annual savings (Figure 3) and a cumulative reduction of $243 (SE $38.5) billion in CMS healthcare expenditures between 1989 and 2008.

#### Out-of-sample forecasts

The out-of-sample forecasts using the model estimated in this paper that uses current smoking prevalence and cigarette consumption per smoker as the measure of smoking behavior performs better than the previously estimated model that used per capita cigarette consumption. The new model performs better on all forecast performance measures, particularly for per capita cigarette consumption. (See Table S1 in the Supporting Information S1 for the results of out of sample forecasts).

## Discussion

The results show that the California Tobacco Control Program had a substantial effect on both smoking prevalence and cigarette consumption per smoker, and both in turn had a substantial effect on per capital healthcare expenditure. The out-of-sample forecasts of the model (using the CMS measure of healthcare expenditure) presented in this study using prevalence and cigarette consumption per smoker are superior to the previously published model that uses per capita cigarette consumption.

From 1989 to 2008, the California Tobacco Control Program cost $2.4 billion and resulted in $243 billion (SE $38.5 billion) in CMS health expenditure savings by reducing total cigarette consumption by a total of 6.79 billion (SE 0.605 billion) packs of cigarettes worth $28.5 billion (SE $2.55 billion) in pre-tax sales to the tobacco industry. 36.4% (SE 4.06%) of this effect was due to reductions in prevalence and 63.6% (SE 4.06%) was due to reductions in consumption among continuing smokers. The fact that such a large fraction of the total effect was due to reductions in consumption points to the importance of considering per smoker consumption in addition to changes in prevalence when evaluating the effects of tobacco control programs. The California Tobacco Control Program has been shown in other research to reduce the prevalence of heavy (>20 cigarettes per day) and moderate smoking (10 to 19 cigarettes per day), and increase the prevalence of light (<10 cigarettes per day) smoking [28], [29].

### Comparison with Existing Estimates

The estimated NIPA healthcare expenditures attributable to smoking using the new model are $548 (SE $27.8) per capita and between $2,262 (SE $121) and $2,904 (SE $184) per smoker. About one third of the smoking-related cost is due to smoking prevalence and the rest due to consumption per smoker.

The estimated annual per capita excess per capita healthcare expenditure (using the CMS measure) attributable to differences in per capita cigarette consumption in our earlier paper [1] was $1,154, which is consistent with $4,910 (SE $373) and $5,982 (SE $411) per smoker, estimated over the sample period 1980 to 2004. Using the new model in this paper, the per capita healthcare expenditure (CMS measure) attributable to an additional smoker who smokes the average number of cigarettes per year as other smokers is $949 (SE $173) per capita, and consistent with between $3,968 (SE $727) and $5,108 (SE $957) per current smoker, which are similar to our earlier paper. NIPA is a better source of healthcare expenditure data for statistical time series analysis because it omits some expenditures categories that are low quality, for example, drug expenditure data for which actual nationally representative survey data are not available for all years. The CMS measure is more comprehensive and more commonly used to measure the burden of healthcare expenditure in the US. The two measures are highly correlated, but the measured per capita expenditures differ in levels [30], [31].

The cumulative reduction in packs sold attributable to the California Tobacco Control Program (between 1989 and 2004) is 4.2 (95% CI 3.4, 4.9) million packs, which is not significantly higher than the 3.6 (95% CI 1.5, 5.9) million packs estimated in using our previous model [1] (P = 0.63 assuming normality). This nonsignificant difference may be due to the use of per capita cigarette consumption in the old model, which included a deterministic time trend [1], and underestimated the reduction in packs consumed attributable to the Program (the new model avoided the need to introduce a time trend). Recursive estimates, starting in 1985, of the old per capita model showed that the coefficient for tobacco control funding increased, while the time trend coefficient approached zero and became statistically insignificant; corresponding recursive estimates of the new model were stable over different subsamples. The new model with prevalence and consumption per smoker is more stable over different sample periods, and therefore we believe more reliable, than the old model using per capita consumption. Our earlier per capita model may have underestimated the effect of California Tobacco Control Program funding on both smoking behavior and healthcare expenditure because the California Tobacco Control Program affects prevalence and cigarette consumption per smoker differently; estimates of program effect that use per capita cigarette consumption is a poorer approximation than using prevalence and consumption per smoker.

The average price elasticity over the sample period of prevalence is −0.198 (SE 0.0951) and of cigarette consumption per smoker is −0.352 (SE 0.164). The total elasticity of cigarette demand is −0.474 (SE 0.164). The results are more consistent with existing price elasticity estimates for cigarette demand [8] than the old model using per capita cigarette consumption, so the new model is more consistent with existing estimates of demand.

The VAR regression approach used in this study is consistent with the cointegrating regression estimates in previous research, and produces a similar long run relationship as the cointegrating regression approach. The prevalence of smoking may be stationary with high autocorrelation, or nonstationary with a unit root. If the data are nonstationary, then the dynamic VAR equations can be solved estimate the combined cointegrating equation and error correction model that should equal the static cointegrating regressions. If the data are stationary, but with high autocorrelation, the VAR estimates are still consistent; the consistency of the static cointegrating regressions can be questioned. Thus, the VAR are more robust if the data are really stationary, and will give the same result for the long relationships as the cointegrating regressions if the data are nonstationary.

### Limitations

This analysis uses aggregate measures of population characteristics to estimate the relationships between per capita tobacco control funding, smoking and per capita healthcare expenditures. The estimated relationship between smoking and healthcare expenditures reflects differences in smoking behavior and healthcare expenditures in different state populations with different histories of aggregate population measures of smoking and resulting cost estimates should not be interpreted as healthcare costs arising in, or due to, an individual smoker. These estimates reflect all the healthcare expenditures associated with smoking that will arise in a population: short and long term direct effects on the smoker, and short and long term effects of second- and third-hand [32] smoking exposure in nonsmokers, not just the effects of smoking on the individual smoker.

The results of this study are subject to the limitations of analysis of aggregate observations using observational data. A study of this nature that used aggregate data and a relatively small sample size cannot, by itself, establish a causal connection between tobacco control programs, smoking behavior and healthcare costs, and is not the goal of this study. Rather, it should be evaluated in the context of the existing body of research that has already established that this relationship is causal using a variety of study designs [33], [34], [35], [36]. There is also a well-established causal relationship between smoking behavior and healthcare costs [13]. It is not currently known if or when the net effect of reduced healthcare expenditures due to fewer smokers might be outweighed by increased expenditures due to longer lived nonsmokers, though our estimates indicate that after more than 25 years of reduced smoking in California compared to the rest of the U.S., reduced smoking was associated with lower per capita healthcare expenditures, and 25 years is a long time horizon for many policy decisions.

The best regression specification for cigarette consumption per smoker (Equation 2) is uncertain given the relatively small number of available annual observations; however, the specification search using Autometrics was unable to identify a specification that was clearly superior to that used for the main analysis. The alternative specification chosen by Autometrics for cigarette consumption per smoker contained California tobacco control funding is a statistically significant explanatory variable, consistent with the hypothesis that tobacco control funding reduced consumption in continuing smokers. Therefore, we are confident that tobacco control funding belongs in the regression, despite uncertainty about other aspects of the specification.

Data were not available to conduct a detailed analysis of the possible independent effect of regional variations in local smokefree policies or sales regulations for tobacco on smoking behavior. However, existing research has shown that these factors should be considered intermediating variables for the effects of large scale state tobacco control programs, which operate, in part, through such changes in state tobacco control policy [7]. Therefore simply including them in a single regression specification would produce a downwardly biased estimate of the effect of the state Program.

Omission of exogenous trends that play no intermediating role in determining smoking behavior or healthcare expenditures could produce bias in the estimated regression coefficients. Examples are prevalence of obesity, abusive alcohol consumption, diabetes, prevalence of racial and ethnic populations, regional capacity of healthcare providers, and penetration of managed care organizations. An extensive sensitivity analysis of the possible effect of these factors, reported in previous research for California [7] showed that they did not have a noticeable effect on the results [1].

### Conclusions

The results extend previous results for California [1] that used per capita cigarette consumption to measure smoking behavior to a similar model that uses a two dimensional measures of smoking behavior: prevalence of smoking and cigarette consumption per smoker. The results indicate that the California Tobacco Control Program was effective in reducing both prevalence of smoking and average cigarette consumption per smoker, and that both measures of smoking behavior have a significant relationship to per capita healthcare expenditures.

Because of the study design, the coefficients for prevalence and consumption per smoker for the health expenditure (Equation 3) cannot identify healthcare costs to smokers themselves due to direct smoking versus costs to others from second and third hand passive smoking, and cannot be used to evaluate the comparative importance of smoking status versus consumption in an individual smoker. The effects of reduced passive smoking due to lower prevalence and consumption may be more important than previously thought: a meta-analysis estimated substantial reductions in hospital admissions for coronary events, other heart disease, stroke, and respiratory disease attributable to increased protection against passive smoking exposure [37], which may partly explain the quick effect of variations in smoking behavior on per capita healthcare expenditure.

The results suggest that tobacco control is very effective at reducing consumption in smokers in addition to reducing prevalence, and that reduction in consumption in continuing current smokers also makes an important contribution to reducing healthcare expenditure for the overall population. Tobacco control programs should evaluate their effectiveness using both changes in prevalence and consumption in current smokers. At the same time, since even low levels of cigarette consumption substantially increase the risk of some diseases, particularly cardiovascular disease [38], [39], [40], [41], [42], [43], [44], eliminating tobacco use should be the ultimate goal.

## Supporting Information

Supporting Information S1 **Details of data sources, modeling methods and sensitivity analysis.**
(DOCX)

Table S1 **Out of sample forecast performance measures for models with alternative measures of forecast performance.**
(DOCX)
