# Supplementary material for: The Effect of the California Tobacco Control Program on Smoking Prevalence, Cigarette Consumption, and Healthcare Costs: 1989–2008
Source: PLoS One. 2013 Feb 13;8(2):e47145. doi: 10.1371/journal.pone.0047145 (PMC3572143; doi:10.1371/journal.pone.0047145)
Supplement: Supporting Information S1 — Details of data sources, modeling methods and sensitivity analysis. (DOCX) [file pone.0047145.s001.docx]

**SUPPORTING INFORMATION S1: DETAILS OF DATA SOURCES, MODELING METHODS AND SENSITIVITY ANALYSIS**

For **The Effect of the California Tobacco Control Program on Smoking Prevalence, Cigarette Consumption, and Healthcare Costs: 1989-2008**

James Lightwood^*^

Stanton A. Glantz^**^

^*^ School of Pharmacy and Center for Tobacco Control Research and Education, University of California, San Francisco, California, United States of America

^**^ Department of Medicine (Cardiology), Center for Tobacco Control Research and Education, and Philip R. Lee Institute for Health Policy Studies, University of California, San Francisco, California, United States of America

Corresponding author:

James Lightwood

Department of Clinical Pharmacy

School of Pharmacy

3333 California Street, Suite 420

San Francisco, CA 94118

Phone: 415-514-0939

Fax: 415-502-0792

E-mail: [lightwoodj@pharmacy.ucsf.edu](mailto:lightwoodj@pharmacy.ucsf.edu)

**Data**

Prevalence of current smoking was from the Behavioral Risk Factor Surveillance System (BRFSS) as provided by the CDC State System [1]. Per capita cigarette consumption was from the *Tax Burden on Tobacco*, as provided by the CDC State System [1]. Cumulative tobacco control funding was calculated from databases provided by the Campaign for Tobacco Free Kids [2] and the CDC State System [1] which were checked for consistency where the same funding elements appeared in both sources. The definition of tobacco control funding used for the main analysis included state and federal funding. The estimates differ slightly from the previous analysis for California [3], which was completed before comprehensive funding records were available from the CDC, and which included only state funding for the California program, which accounted for over 99 percent of the California Tobacco Control Program [1,2]. Private funding was omitted, though including it makes almost no difference in the results. The price per pack of cigarettes was from the *Tax Burden on Tobacco*, as provided by the CDC State System [1].

The National Income and Product Account (NIPA) measure of per capita healthcare expenditure is from the U.S. Bureau of Economic Analysis [4]. The Centers for Medicare and Medicaid Services (CMS) measure of healthcare expenditure is from the CMS [5,6]. The NIPA measure omits components from lower quality sources, such as prescription drug expenditures (which are interpolated data from surveys conducted every five years without using any information from annual surveys) and so is more appropriate to use for statistical analysis than the CMS measure [7,8]. The two measures of healthcare expenditure are highly correlated over time, and both include expenditures for hospital services, medical procedures and healthcare personnel [9]. Total resident population data was from the U.S. Census Bureau [10,11,12]. Proportion of the population by race and ethnicity, used for sensitivity analysis, was calculated from the BRFSS data [13] rather than Census data because complete data using consistent definitions were not available from the U.S. Census over the whole sample period, and the model effects of annual Census population estimates by race and ethnicity are so large that they cannot be used in regression analysis without introducing spurious results due to deterministic trends between census years. The aggregate control population variables were population weighted [10,11,12], except for cigarette prices [1] which were cigarette sales weighted.

**Statistical Analysis**

The analysis used a reduced form vector autoregression (VAR) specification in which the dependent variable is expressed as a function of lagged explanatory variables, which can be used for a wide variety of stationary and nonstationary time series [14,15].

Tests for unit roots in the variables and regression residuals were conducted in order to guide regression model specification, choice of estimation method and interpretation of the results using the Phillips-Perron [16] and KPSS [17] unit root tests.

Equations 1 to 3 were estimated using instrumental variables with artificially generated trending irrelevant instrumental variables (IIV) technique [18,19,20] and Heteroskedastic Autocorrelation Consistent (HAC) [21] standard errors. The IIV estimates of the VAR specification were not adjusted for unit root bias due to the resulting small degrees of freedom, though in a sensitivity analysis the estimates were adjusted in order to determine any difference between the two estimators. The behavior of the regression residuals was checked for normality, serial correlation, heteroskedasticity, influential outliers and structural breaks [22].

**Estimated Program Effect**

*Details of Calculation of Program Effect in Monte Carlo Simulation*

A Monte Carlo simulation was done to estimate the program effect by comparing the distribution of smoking behavior and per capita healthcare expenditures by comparing the observed historical time series of tobacco control funding in California and control states to an alternative time series where tobacco control funding in program was set to zero over the sample period. Details of the simulation are given below.

The predicted historical time series were described with the estimated versions of Equations 1 through 3 from the main text:

*Current Smoking Prevalence:*

(A1)

*Cigarette Consumption per Smoker:*

(A2)

*Current Smoking Prevalence, Cigarette Consumption per Smoker and Healthcare Expenditures:*

(A3)

(A4)

Note that Equations A1, A2 and A3 are simply the estimated regression equations corresponding to Equations 1, 2 and 3 in the main text, and which are reproduced below for reference. Equation A4 is corresponds to the equation for per capita healthcare expenditure in the text (Equation 3), except that the CMS measure of per capita healthcare expenditure is used instead of the NIPA measure.

*Current Smoking Prevalence:*

$(prev_{c, t}-prev_{CA, t})=\alpha_{0}+\alpha_{1}(EC_{CA, t-1}-EC_{c, t-1})+\alpha_{2}(p_{c, t-1}-p_{CA, t-1})+\alpha_{3}(y_{c, t-1}-y_{CA, t-1})+\varepsilon_{1,t}$ (1)

*Cigarette Consumption per Smoker:*

$(cps_{c, t}-cps_{CA, t})=\beta_{0}+\beta_{1}(EC_{CA, t-1}-EC_{c, t-1})+\beta_{2}(p_{c, t-1}-p_{CA, t-1})+\beta_{3}(y_{c, t-1}-y_{CA, t-1})+\varepsilon_{2,t}$ (2)

*Current Smoking Prevalence, Cigarette Consumption per Smoker and Healthcare Expenditures:*

$n_{CA, t}=\gamma_{0}+\gamma_{1}n_{c, t}+\gamma_{2}(prev_{c, t-1}-prev_{CA, t-1})+\gamma_{3}(cps_{c, t-1}-cps_{CA, t-1})+\gamma_{4}(y_{c, t-1}-y_{CA, t-1})+\varepsilon_{3,t}$ (3)

For reference in this Appendix, we write out the equation for healthcare expenditure using the CMS measure of healthcare expenditure below:

$h_{CA, t}=\eta_{0}+\eta_{1}h_{c, t}+\eta_{2}(prev_{c, t-1}-prev_{CA, t-1})+\eta_{3}(cps_{c, t-1}-cps_{CA, t-1})+\eta_{4}(y_{c, t-1}-y_{CA, t-1})+\varepsilon_{4,t}$ (3a)

The counterfactual of no California Tobacco Control Program funding was computed by setting and $\hat{\beta}_{1}$ to 0 in Equations 1 and 2, while leaving all the other estimates for the coefficients the same as estimated from the full data series. Specifically,

*Current Smoking Prevalence:*

(A5)

*Cigarette Consumption per Smoker:*

(A6)

*Current Smoking Prevalence, Cigarette Consumption per Smoker and Healthcare Expenditures:*

(A7)

(A8)

where

, from equations A1 and A5

, from Equations A2 and A6

$x$ = *h* for historical simulated time series, and *a* for alternative no funding simulated time series.

The parameters with hats and $e_{1,t}, e_{2,t}, e_{3,t}, e_{4,t}$are the estimated regression residuals from regression estimates for Equations 1, 2 and 3 for the NIPA measure of healthcare expenditure; and for Equations 1, 2 and 3a for the CMS measures of healthcare expenditure in the main text (Table 1). The distributions of these residuals were used in the simulations to calculate confidence intervals for the estimates.

The distributions for the difference between the historical and alternative no funding cases were estimated by simulating the system A1 to A8 above for t = 1989 to 2008, using one random draw for the parameter standard errors for all years and one random draw for the regression residuals for each year for each trial in the Monte Carlo simulation. Years 1989 to 1990 were used to initiate the simulated time series due to lags in the equation system. Therefore, the first difference between historical and alternative year occurs in 1991 for current smoking prevalence and mean cigarette consumption per smoker, and in 1992 for per capita healthcare expenditures. The pattern of the first nonzero differences in the simulation estimates are, again, due to the pattern of lags in Equations A1 to A8.

The estimated parameters are normally distributed with one value drawn for each trial of the Monte Carlo simulation. The regression errors for Equations 1 to 4, respectively, were simulated using random draws for $e_{1,t}, e_{2,t}, e_{3,t}, e_{4,t}$, drawn from normal distributions with mean zero and variances estimated from estimated variances of the regression errors for Equations A1, A2, A3 and A4 above, respectively. The random variates are drawn from independent normal distributions with means equal to the estimated regression parameters with standard deviations equal to the standard errors of the corresponding regression estimates in Equations A1 and A2 ; are drawn from a bivariate normal distribution with variances and covariance from the estimated variance-covariance matrix for the regression coefficients for Equation A3; are drawn from a bivariate normal distribution with variances and covariance from the estimated variance-covariance matrix for the regression coefficients for the estimated version of Equation 3a above.

The predicted smoking prevalence$(prev_{CA, t})$ and consumption per smoker $(cps_{CA, t})$and measures of per capita healthcare expenditure$(n_{CA, t}\text{and }h_{CA, t})$ for California were calculated by subtracting the observed values for control states from the dependent variables calculated in Equations A1 to A8 in each Monte Carlo trial. Person years of smoking for California in each year was calculated by multiplying the simulated prevalence by the resident adult population, where adult is defined as age 18 years or older in California in each year. The estimate for total packs of cigarettes consumed in California was calculated by multiplying adult prevalence times mean cigarette consumed per current smoker times adult population in California for each year. Differences attributable to the California Tobacco Control Program were taken by calculating the differences between the predicted dependent variables in Equations A1 and A5, between A2 and A6, between A3 and A7, and between A4 and A8 for each of the Monte Carlo trials. Differences between total packs of cigarettes consumed and person years of smoking were calculated by taking the differences between the predicted value with the historical tobacco control program, and with the history of tobacco control funding set to zero. The simulation used 20,000 trials.

**Sensitivity Analysis**

*Validation of model specification using a specification search algorithm*

Autometrics [22] is an automatic model selection algorithm that chooses the best model specification from a set of explanatory variables by using a series of tests of the null hypothesis that the coefficient of an explanatory variable is zero (that is, whether an explanatory variable should be included in the regression specification) that preserve the validity of the final variance estimates for statistical inference after screening multiple specifications. Autometrics also screens for acceptable performance of regression residuals and discards specifications that produce residuals that indicate misspecification of the regression equation, using the Schwartz information criterion as the final selection criterion where no single candidate specification dominates the others [22,23].

The Autometrics algorithm was run separately for each equation in the model using the dependent variables in Equations 1 to 3 and also including additional lags of the explanatory variables, and additional potential explanatory variable for age structure (proportion of population elderly) because we found age structure to be an important variable in our analysis of the effects of the Arizona tobacco control program [24]. A maximum of three annual lags were used for the explanatory variables in the VAR specification, due to small degrees of freedom when more lags were included. Autometrics found that one lag is sufficient for reduced form VAR specifications. A specification that included a variable that describes the age structure of the population was dropped because regression specifications with age did not perform as well as others, and its inclusion or exclusion did not noticeably affect the estimates of the other coefficients.

No specification search was done for the healthcare expenditure equation using the CMS measure (Equation 3a); the specification for the NIPA healthcare expenditure was used. This approach to specification produces a more rigorous test for out of sample performance of the new specification using smoking prevalence and cigarette consumption per smoker compared to the previously published per capita cigarette consumption model using the CMS measure of healthcare expenditure.

*Use of alternative estimators*

If the time series of the variables contain autoregressive unit roots, then their long run relationship can be estimated directly using a static linear regression specification called a cointegrating regression [14,25], as we did in our earlier model of California [3]. The cointegrating regression can be derived for each of Equations 1 to 3 by simply setting the time subscript *t*-1 to *t* for the explanatory variables. A static cointegrating regression specification corresponding to Equations 1 to 3 was estimated using OLS and IIV using heteroskedastic Autocorrelation Consistent (HAC) [21] standard errors. The IIV estimates were estimated with and without adjustment for unit root bias. The residuals were checked for stationarity using the same procedures as for the unit root tests. When there was evidence of influential observations, robust regression estimates were calculated to check the results when influential observations were given less weight. The results of these regressions were compared to the results using a reduced form VAR specification to check for consistency.

*Alternative specification for consumption per smoker*

The analysis was redone using an alternative regression model for cigarette consumption per smoker (Equation 2) suggested by the Autometrics specification search. The alternative specification modeled California consumption per smoker as a function of only three variables: the difference between California and control cumulative tobacco control funding, and cigarette price in California.

*Race and Ethnicity*

The model was re-estimated with variables for racial and ethnic composition of California and control populations, using estimates of the proportion of white, black and all other races from BRFSS survey data, added to Equations 1 to 3 to in order to determine the sensitivity of the regression estimates to these population characteristics. Race and ethnicity were not included in the model specification search algorithm because reliable estimates of race and ethnicity were not available from the BRFSS for the whole sample period due to small numbers of respondents for some categories in the early years of the survey. Estimates of the proportion of the population by race and ethnicity using Census data were not used because the annual estimates, which are estimated using a demographic model, approximated deterministic trends over the intervals with interpolated data and introduced autoregressive artifacts and multicollinearity into the estimates.

**Estimates using Centers for Medicare and Medicaid Services (CMS) Healthcare Expenditure Data**

CMS provides two sets of state-level data, one based on location of healthcare provider (first year of data is 1980) and one based on residence of patient (first year of data is 1991). CMS recommends that only the state data by residence be used for per capita expenditure estimates [7,8]. When aggregated for this analysis, the two series are almost identical and yield almost identical results. Therefore, for this analysis the state provider data were used because that series has a larger sample size and the estimates are therefore more precise.

**Out of sample forecasts of the CMS measure for healthcare**

The regression specification used for per capita healthcare expenditure using the NIPA measure of healthcare expenditure (Equation 3) was used for the specification for per capita healthcare expenditure using the CMS measure (Equation 3a). Equation 3a was estimated using data for the years 1984 to 2004, and these estimates were used to generate out of sample forecasts for the period 2005 through 2008. Equation 2 from previous research [3] was used to generate corresponding out of sample estimates over the same period. The two out of sample forecasts were compared using common measures of forecast performance: root mean square error (RMSE), mean absolute error (MAE), mean absolute percent error (MAPE), and a regression of the forecast on observed values for each model.

**RESULTS**

**Model Estimates**

*Residual diagnostics*

The residuals for all three regressions were stationary, homoskedastic, and showed no statistically significant autocorrelation, except for prevalence (Equation 1) that showed some fourth and higher order autocorrelation. The residuals for the health expenditure regression (Equation 3) were non-normal because of two potentially influential outliers, though the sensitivity analysis showed that they did not affect the estimates.

**Sensitivity Analysis**

*Alternative estimators and control states*

The results of the OLS estimates of the VAR and cointegrating regressions consistent with those of the reduced form VAR estimates. Static regression specifications that represented the long run relationships were cointegrating (the residuals were stationary) and both the adjusted and unadjusted IIV estimates of the cointegrating regression for Equations 1 to 3 were similar to those of the reduced form VAR specification. Robust estimates, which reduced the effect of outliers and influential observations, of the reduced from VAR and cointegrating regressions did not differ substantially from the least squares estimates.

*Alternative Selection of Control States*

The estimates for Equations 1 to 3 using alternative control populations were similar to the main results. For prevalence (Equation 1), the coefficients for tobacco control funding, , ranged from 0.0428 (SE 0.00294) percentage points prevalence per dollar of cumulative per capita tobacco control funding using the rest of U.S. as control to 0.0635 (SE 0.00502) percentage points prevalence using equally weighted 13 control states. For consumption per smoker (Equation 2), the coefficient for tobacco control funding, , ranged from 1.18 (SE 0.0705) packs per smoker per dollar of cumulative per capita tobacco control funding for Western Region to 1.91 (SE 0.118) packs per smoker for equally weighted 13 control states. For per capita healthcare expenditures (Equation 3), the coefficients for prevalence, , ranged from $42.6 (SE $15.2) per prevalence point of smoking prevalence for the rest of the U.S. to $53.5 (SE $8.91) per prevalence point of smoking prevalence for equally weighted 13 control states; for cigarette consumption per smoker, ,the coefficients ranged from $0.920 (SE $0.283) packs per smoker for the Western Region to $3.87 (SE $0.306) packs per smoker for equally weighted 13 control states.

*Alternative specification of consumption per smoker*

The estimated coefficients in Equation 2 using the alternative model chosen by Autometrics were -2.96 (SE 0.232) for the difference California and control state tobacco control funding and -15.46 (SE 5.00) for the price of cigarettes in California. Tobacco control funding has a statistically significant effect on cigarette consumption per smoker in California in the alternative model.

*Race and Ethnicity*

The variables for proportion of the population that African-American or Hispanic did not enter the regressions (all P values > 0.10) and their inclusion did not change the values of the other coefficients substantially. The variable for Other Race (neither White nor African-American) did enter the regressions for prevalence (Equation 1) and cigarettes consumption per smoker (Equation 2) at the 5 percent significance level, and slightly increased the tobacco control funding coefficients, $\alpha_{1}$ and $\beta_{1}$, in both equations; however, the estimates for these coefficients were not significantly different from those shown in the main text at the 5 percent significance level.

**Centers for Medicare and Medicaid Services (CMS) Healthcare Expenditure**

Using the CMS measure of health expenditure (rather than the NIPA measure), a one percentage point reduction in prevalence of current smoking and consumption of one pack per year per smoker in California reduces per capita healthcare expenditures by $69.8 (SE $12.6) and $5.59 (SE $1.77), respectively. Using the CMS measure of healthcare expenditure (rather than the NIPA measure); California tobacco program was associated with a cumulative reduction of $142 (SE $22.4) billion in healthcare expenditures between 1989 and 2004. Estimates of healthcare expenditure using the CMS measure of healthcare expenditure (rather than the NIPA measure) from 1985 to 2008, a one percentage point reduction in prevalence of current smoking and consumption of one pack per year per smoker in California reduces per capita healthcare expenditures by $67.8 (SE $7.31) and $5.48 (SE $0.928), respectively. The California Tobacco Control Program was associated with a cumulative reduction of $243 (SE $38.5) billion in healthcare expenditures between 1989 and 2008.

*Out-of-sample forecasts*

The out-of-sample forecasts using the model estimated in this paper that uses current smoking prevalence and cigarette consumption per smoker as the measure of smoking behavior performs better than the previously estimated model that used per capita cigarette consumption. The new model performs better on all forecast performance measures, particularly for per capita cigarette consumption. The results of the out of sample forecasts are shown in Table S1.

**REFERENCES**

1. Centers for Disease Control and Prevention (CDC) (2011) State Tobacco Activities Tracking and Evaluation (STATE) System. Centers for Disease Control and Prevention.

2. Tobacco Free Kids (2011) U.S. State and Local Issues: A Broken Promise to Our Children. Tobacco Free Kids.

3. Lightwood JM, Dinno A, Glantz SA (2008) Effect of the California tobacco control program on personal health care expenditures. PLoS Medicine 5: e178.

4. Bureau of Economic Analysis (BEA) (2011) State Annual Personal Income and Employment 1929–2010. Bureau of Economic Analysis.

5. Centers for Medicare and Medicaid Services (2011) Health Expenditures by State of Provider, 1980-2009 (compressed excel file). U.S. Department of Health and Human Services.

6. Centers for Medicare and Medicaid Services (CMS) (2011) Health expenditures by state of residence, 1991-2009 (compressed excel file). U.S. Department of Health and Human Services.

7. Centers for Medicare and Medicaid Services (2011) State Health Expenditure Accounts: State of Provider Definitions and Methodology, 1980-2009 (PDF file). U.S. Department of Health and Human Services.

8. Centers for Medicare and Medicaid Services (CMS) (2011) Health Spending by State of Residence, 1991–2009 (PDF file). U.S. Department of Health and Human Services.

9. Kornfeld R (2011) Health Care Expenditures in the NHEA and GDP. National Economic Accounts Data Users Conference. Washington DC: Bureau of Economic Analysis. pp. 1-17.

10. United States Census Bureau (2004) State resident population by age and sex, 1980-1989. United States Bureau of the Census.

11. United States Census Bureau (2004) State resident population by age and sex, 1990-1999. United States Bureau of the Census.

12. United States Census Bureau (2011) Intercensal Estimates of the Resident Population by Single Year of Age and Sex for States and the United States: April 1, 2000 to July 1, 2010. United States Bureau of the Census.

13. Centers for Disease Control and Prevention (CDC) (1984-2009) Behavioral Risk Factor Surveillance System Survey (BRFSS) Data. Atlanta, Georgia: U.S. Department of Health and Human Services, Centers for Disease Control and Prevention.

14. Enders W (2004) Applied Econometric Time Series. Hoboken, NJ: John Wiley and Sons.

15. Maddala GS, Kim I-M (1998) Unit Roots, Cointegration, and Structural Change. Cambridge: Cambridge University Press.

16. Phillips P, Perron P (1988) Testing for a unit root in time series regression. Biometrika 75: 335-346.

17. Kwiatkowski D, Phillips PCB, Schmidt P, Shin Y (1992) Testing the null hypothesis of stationarity against the alternative of a unit root: how sure are we that economic times series have a unit root? Journal of Econometrics 54: 159-178.

18. Kourogenis K, Panopoulou E, Pittis N (2005) Irrelevant but Highly Persistent Instruments in Stationary Regressions with Endogenous Variables Containing Near-to-Unit Roots. Piraeus, Greece: Department of Banking and Financial Management, University of Piraeus.

19. Phillips PCB (2006) Optimal estimation of cointegrated systems with irrelevant instruments. New Haven, CT: Cowles Foundation, Yale University.

20. Phillips PCB, Hansen BE (1990) Statistical inference in instrumental variables regression with I(1) processes. The Review of Economic Studies 57: 99-125.

21. Andrews D (1991) Heteroskedasticity and autocorrelation consistent covariance matrix estimation. Econometrica 59: 817-858.

22. Doornik J, Hendry D (2009) Empirical Econometric Modelling, PC Give 13, vol I. Timberlake Consulting, Ltd.: London, UK.

23. Doornik J (2009) Autometrics. In: Castle J, Shephard N, editors. The Methodology and Practice of Econometrics. Oxford, UK: Oxford University Press. pp. 88-121.

24. Lightwood J, Glantz S (2011) Effect of the Arizona tobacco control program on cigarette consumption and healthcare expenditures. Social Science and Medicine 72: 166-172.

25. Engle RF, Granger C (1987) Co-Integration and error correction: representation, estimation, and testing. Econometrica 55: 251-276.
