# Supplementary material for: The Effect of the California Tobacco Control Program on Smoking Prevalence, Cigarette Consumption, and Healthcare Costs: 1989–2008
Source: PLoS One. 2013 Feb 13;8(2):e47145. doi: 10.1371/journal.pone.0047145 (PMC3572143; doi:10.1371/journal.pone.0047145)
Supplement: Table S1 — Out of sample forecast performance measures for models with alternative measures of forecast performance. (DOCX) [file pone.0047145.s002.docx]

Table S1.-- Out of sample forecast performance measures for models with alternative measures of forecast performance.

|  | Measures of forecast error | | | Regression of forecast values on observed values | |
| --- | --- | --- | --- | --- | --- |
|  | RMSE | MAE | MAPE | Slope coefficient | Standard error |
| Per capita cigarette consumption | | | | | |
| Old model* | 20.2 packs | 6.14 packs | 42.9% | 4.01 | 0.894 |
| New model | 6.69 packs | 3.57 packs | 27.3% | 1.85 | 0.253 |
| Per capita health care expenditure | | | | | |
| Old model* | $168 | $77.4 | 1.23% | 1.63 | 0.578 |
| New model | $155 | $52.8 | 0.841% | 1.51 | 0.611 |

RMSE: Root mean square error; MAE: mean absolute error; MAPE: mean absolute percentage error.

Old model: estimates from previous research [3] using per capita cigarette consumption as the measure of smoking behavior.

New model: estimates from model in this paper that uses current smoking prevalence and cigarette consumption per smoker as the measure of smoking behavior.

Estimation period is 1985 to 2004. Forecast period is 2005 to 2008.

^*^Lightwood, J.M., A. Dinno, and S.A. Glantz, Effect of the California tobacco control program on personal health care expenditures. PLoS Medicine, 2008. 5(8): p. e178.

______________________________________________________________________________
